# Machine and deep learning models for ligament injury recognition: a systematic review and meta-analysis of imaging and novel diagnostic techniques

**DOI:** 10.1530/EOR-2025-0038

**Published:** 2026-01-09

**Authors:** Guillermo Droppelmann, Emilia Varas, Joaquín Villagrán, Carlos Jorquera, Felipe Feijoo

**Affiliations:** ^1^ Clínica MEDS, Santiago, RM, Chile; ^2^ Facultad de Medicina, Universidad de los Andes, Santiago, RM, Chile; ^3^ Facultad de Ciencias, Escuela de Nutrición y Dietética, Universidad Mayor, Santiago, RM, Chile; ^4^ School of Industrial Engineering, Pontificia Universidad Católica de Valparaíso, Valparaíso, Chile

**Keywords:** artificial intelligence, deep learning, diagnostic, ligament, machine learning

## Abstract

**Purpose:**

- Diagnosing ligament injuries remains a challenge for musculoskeletal clinicians due to the lack of standardized classification, evaluation, and management protocols. Machine learning (ML) and deep learning (DL) models offer potential to improve diagnostic accuracy. This study aimed to evaluate the diagnostic performance of various ML and DL models in identifying ligament injuries across different medical imaging modalities.

**Methods:**

- A meta-analysis was conducted following the PRISMA 2020 checklist. Searches were performed in PubMed, SCOPUS, Web of Science, and the Cochrane Library. Study quality was assessed using the QUADAS-2 tool and Robvis software. Diagnostic performance measures – true positive, true negative, false positive, and false negative – were analyzed. A random-effects model was applied, and heterogeneity and subgroup analyses were conducted. Statistical and graphical analyses were performed using R. The study was registered in PROSPERO (CRD42025646317).

**Results:**

- Fifty-nine ML and DL algorithms from 23 studies were analyzed. Pooled sensitivity and specificity were 0.890 (95% CI: 0.829–0.938) and 0.926 (95% CI: 0.820–0.959), respectively. Pooled estimates for PLR, NLR, lnDOR, and AUC were 1,644.37 (95% CI: 73.56–3,215.18), 0.179 (95% CI: 0.095–0.263), 4.130 (95% CI: 3.570–4.700), and 95%, respectively, with *P* < 0.001.

**Conclusion:**

- ML and DL models demonstrate high diagnostic accuracy in detecting ligament injuries. Their strong performance supports ongoing integration into clinical practice, offering valuable support for musculoskeletal specialists in image interpretation and diagnosis.

## Introduction

Modern society has witnessed the unprecedented rise of artificial intelligence (AI), rapidly transforming numerous industries, including transportation, commerce, telecommunications, agriculture, and forest (1). This technological revolution has profoundly impacted medicine and healthcare. Various medical specialties have benefited from AI-driven tools and innovations, enhancing multiple processes and ultimately improving patient outcomes and quality of life (2).

The field of diagnostics has made significant advancements, emphasizing its role in enhancing precision, speed, and decision-making support for healthcare professionals. Both machine learning (ML) models and deep learning (DL) algorithms have contributed to processing large volumes of data (3). Moreover, their remarkable computational power enables the recognition of patterns in medical images that often go undetected by the human eye, sometimes exceeding the precision of specialists (4). Integrating these tools into hospital settings facilitates early detection of disease, allocation of resources, reduced waiting times, and greater diagnostic accuracy. This transformation transforms traditional medicine, which is based on standardized treatments, to precision medicine tailored to the individual needs of the patient (5).

Among medical specialties, musculoskeletal radiology has been one of the most significantly affected by the development and applications of AI. This is due to its strong connection to diagnostic advancements and its dependence on highly specialized technology (6). In addition, the integration of computer vision elements that enhance human–machine interaction has led to rapid adoption by professionals, administrators, and system users. In particular, the implementation of complex computational models capable of analyzing images with high speed and precision – particularly when the region of interest is accurately delineated – has optimized the diagnostic process by reducing interobserver variability and improving reproducibility and reliability in medical image interpretation (7).

In recent years, the use of AI-based strategies for diagnostic support has increased significantly among musculoskeletal radiologists, driven by the advantages mentioned above. This has led to the application of various models and algorithms aimed at optimizing diagnostic performance in medical image analysis (8). These strategies depend on several key factors, including data availability, computational capabilities, hardware requirements, interpretability and explainability, the required level of precision, and, most importantly, the clinical context in which they are applied. As a result, no single method or model is universally superior; instead, the choice must be carefully tailored to these multiple considerations (9).

**Figure 1:**
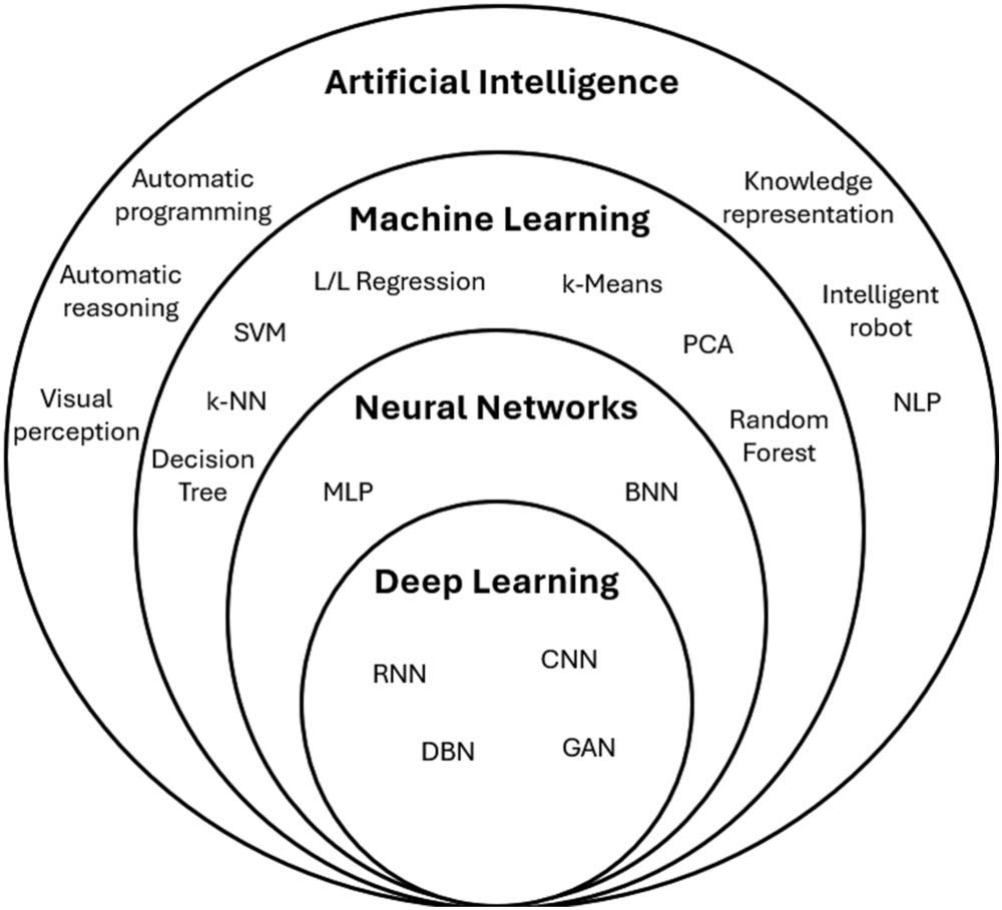
Venn diagram of AI structure.

One possible approach to implementation is to select protocols and methods based on the required level of complexity (10). Simple but effective strategies include deterministic approaches, such as thresholding, logical rules, or statistical metrics for image evaluation, which do not require data training but rely on proper calibration (11). Another widely used approach involves classic supervised and unsupervised learning methods that do not employ deep neural networks. These models can learn patterns from correctly labeled data without explicit human intervention, relying instead on system-fed input (12). A more advanced strategy is to use hybrid methods that combine multiple techniques to improve diagnostic accuracy. These models integrate different approaches to ML and neural networks, leveraging the strengths of each method (13). Finally, the most complex implementation involves convolutional neural networks (CNN) and computer vision models for image processing, which automatically learn by extracting features from individual pixels (14). Figure 1 illustrates the hierarchical relationship of AI.

Ligament injuries remain a key concern in musculoskeletal pathologies due to their high prevalence, disease burden, and significant impact on both general health and quality of life in the general population and athletes (15). In most cases, these injuries result from an initial traumatic event, which, depending on severity, can range from a mild strain to a complete rupture of the affected ligament (16).

The classification of ligament injuries is highly diverse, as it depends on the specific structure being assessed. For example, ultrasound has shown high diagnostic accuracy for ankle sprains (17). In contrast, knee cruciate ligament injuries are easily detected with magnetic resonance imaging (MRI) (18). However, diagnosing ligament injuries in the wrist, hand, and fingers remains controversial (19). Various technological strategies have been developed as tools for detecting the risk of injury in musculoskeletal pathologies. Examples include sensor-based techniques for analyzing movement in sports injuries and portable monitoring systems (20, 21, 22).

Based on our experience, healthcare professionals use different classification systems for these injuries. Some follow a binary approach, distinguishing only between the presence or absence of injury. Others use an ordinal system, categorizing injuries as mild, moderate, or severe based on the degree of ligament distension. Alternatively, a continuous system quantifies damage as a percentage or assesses clinical parameters such as structural alteration and joint stability deterioration.

Regardless of the classification system used, an initial ligament injury often leads to progressive failure over time, increasing the likelihood of recurrent joint instability (23). This condition, in turn, increases the risk of ongoing structural deterioration within the musculoskeletal system, promoting the development of progressive joint damage and, ultimately, secondary osteoarthritis (24). Traditionally, the onset of these injuries has been primarily attributed to trauma. However, recent studies suggest that alterations in specific brain structures involved in motor control and anticipation of biomechanical responses can influence both injury susceptibility and the patient’s ability to recover (25). Therefore, early detection and precise diagnostic evaluation are essential for optimizing therapeutic strategies, preventing further damage to affected structures, and improving long-term clinical outcomes.

Previous studies have demonstrated with high precision the effectiveness of these diagnostic strategies in musculoskeletal pathologies affecting the upper extremity (26), and, more recently, in tendinopathies using various DL models and imaging modalities which have shown a high level of precision in detecting abnormalities (27). However, to the best of the authors’ knowledge, no studies have yet comprehensively analyzed the range of algorithms available in the scientific literature for the detection and classification of ligament injuries. This systematic review and meta-analysis aims to evaluate the diagnostic performance of multiple ML and DL models in recognizing ligament injuries in different medical imaging modalities.

## Methods

### Reporting

This meta-analysis was carried out according to the PRISMA guidelines (Preferred Reporting Items for Systematic Reviews and Meta-Analyses). The 27-item checklist, which ensures comprehensive reporting in the Introduction, Methods, Results, and Discussion sections of a systematic review, was fully verified. This checklist is available at www.prisma-statement.org. Furthermore, the authors voluntarily registered the review on the PROSPERO platform: CRD42025646317.

### Research question

This study aimed to evaluate the diagnostic performance of various ML and DL models in recognizing ligament injuries in different medical imaging modalities and novel diagnostic techniques. The research question was structured using the PICOT framework (participants, intervention, comparison, outcome, and time), which is detailed in Table 1.

**Table 1:** Summary of study components.

Acronym	Component	Explanation
(P)	Population	Patients diagnosed with ligament injury who have any type of diagnostic imaging or novel diagnostic method
(I)	Intervention	Any type of ML and DL model used for diagnostic purposes
(C)	Comparison	Conventional diagnostic method
(O)	Outcome	Evaluate the diagnostic performance of various ML and DL models
(T)	Type of study	Diagnostic study

### Search strategy and data sources

The lead author, GD, a specialist in musculoskeletal injuries, selected keywords related to ligament injuries. The validation of diagnostic terminology was performed by an external collaborator, a radiologist with subspecialty expertise in musculoskeletal disorders. Keywords related to ML or DL were identified and validated by coauthor FF, PhD in engineering. Each member of the professional team has over 15 years of experience.

The PubMed search engine confirmed that all selected terms corresponded to medical subject headings (MeSH). The final terms included in this review were: ligaments, ligament, diagnosis, diagnostic imaging, AI, neuronal network (NN), convolutional neural network (CNN), artificial neural network (ANN), new diagnostic method, and novel diagnostic method. Subsequently, two coauthors, EV and JV, conducted a systematic search in selected databases, including: MEDLINE/PubMed (https://www.ncbi.nlm.nih.gov/pubmed/), WOS/Web of Science (https://clarivate.com/), SCOPUS (https://www.scopus.com/home.uri), the Cochrane Library (https://www.cochranelibrary.com/).

Any discrepancies between the reviewers were resolved by a third independent reviewer, CR. The review covered 10 years, from September 2014 to September 2024. Finally, a comprehensive data matrix was generated by simultaneously combining all possible variations of the selected terms. Full access was obtained to all articles included in this study.

### Selection criteria

#### Inclusion criteria

i) complete and published original scientific articles. ii) Studies focusing on ligament injuries in which diagnosis was supported by ML or DL tools. iii) Original scientific articles that included any type of radiological imaging or novel diagnostic method, regardless of the location of the injury. iv) Original scientific articles that incorporate at least one ML or DL model as a diagnostic method. v) Original scientific articles explicitly reporting true positives (TP), false positives (FP), true negatives (TN), and false negatives (FN) for the calculation of sensitivity and specificity. vi) Original scientific articles explicitly reporting sensitivity, specificity, positive predictive values (PPV), and negative predictive values (NPV). vii) Original scientific articles published in English, Spanish, or Portuguese. viii) Original scientific articles published within the last 10 years, up to September 2024. ix) Human ligaments.

#### Exclusion criteria

i) scientific articles classified as reviews, letters, conference reports, studies using cadaveric samples, or technical descriptions. ii) Studies focused on medical or technological devices, sensors, virtual reality, or any tangible (hardware) or intangible (software) object that does not incorporate ML or DL models.

### Data extraction

A preliminary screening was performed by reviewing titles and abstracts. Original scientific articles in full text that met the predefined selection criteria were then selected, while duplicate manuscripts were removed. A data matrix was created using Microsoft Excel, including the following variables: authors, year of publication, country of origin, number of images used in the validation process, type of imaging modality, clinical diagnostic condition, and computational model used.

The TP, FN, FP, and TN reported for each model were identified. As is customary in diagnostic performance analysis, if an article included multiple models, all reported results were considered, with each model specifically identified by name. In cases in which these metrics were not provided, data on sensitivity, specificity, PPV, and NPV were extracted, along with the respective sample sizes in the validation set. Using all available information, the diagnostic metrics for sensitivity, specificity, precision, and F1 score were calculated. Finally, data extraction was performed manually by two coauthors, EV and JV, under the supervision of a third author, CJ. Any discrepancies were resolved by the lead author.

### Ethical approval

Although specific approval from an ethics committee is not required for conducting a systematic review and meta-analysis, this study included only research that adhered to the universal principles outlined in the Declaration of Helsinki. Furthermore, all selected studies had received approval from a scientific ethics committee and ensured the proper execution of informed consent procedures.

### Risk of bias (quality) assessment

The QUADAS-2 (quality assessment of diagnostic accuracy studies) guidelines were used to evaluate the quality of the selected articles and identify potential biases that could affect the interpretability of the results. The studies were classified into three levels: low, some concerns, or high (28).

The QUADAS-2 tool consists of four key domains that discuss patient selection, index test, reference standard, patient flow through the study, and timing of index tests and reference standard (28). The coauthors jointly reviewed the patient selection methods and assessed whether the index test was clearly described in terms of application and interpretation. The reference standard was analyzed to ensure that its use was explicitly detailed. Finally, it was verified whether all patients underwent both tests and whether any time interval or intervention could have influenced the results.

The graphs were generated using the Robvis application, developed with the Robvis package in R. The structure of the QUADAS-2 example dataset in Excel was used, and the analysis was performed step by step according to the platform guidelines. This tool is freely available at: https://mcguinlu.shinyapps.io/robvis/.

### Statistical analysis: univariate and bivariate methods

To assess the diagnostic performance of the models, TP, FN, FP, and TN were calculated when not explicitly reported, using sensitivity, specificity, PPV, and NPV. Furthermore, the positive likelihood ratio (PLR) and the negative likelihood ratio (NLR) were estimated with their respective 95% confidence intervals (95% CI), considering the relationship between the number of events and the sample size for each model.

To enhance the stability of the results, a logistic transformation followed by an inverse transformation was applied using the Clopper–Pearson method. For better comparison and synthesis of results, the diagnostic odds ratio (DOR) and its logarithmic version (lnDOR) were calculated. Finally, data were visually represented using forest plots. To address the secondary research question, a bivariate analysis was performed, categorizing the model subgroups into three categories according to their architectural complexity.

- **Group 0, (*g* = 0):** low- and moderate-complexity models.
- **Group 1, (*g* = 1):** high-complexity models.

For each subgroup, DOR was calculated and visually represented using a forest plot. In addition, diagnostic accuracy metrics were estimated using the area under the curve (AUC), and a summary receiver operating characteristic (SROC) curve was generated to summarize the overall performance of the models.

### Heterogeneity analysis

A random effects model was applied due to the significant heterogeneity observed among the selected studies. Variability was estimated using the inverse variance method, assigning a specific weight to each study, and the tau (*τ*) value was calculated using the DerSimonian–Laird estimator.

The influence of heterogeneity on total variability, compared to random variability between studies, was assessed using the Higgins *I*^2^ statistics, both for the overall data set and within subgroups. Heterogeneity was classified into four levels: minimum: 0–40%; moderate: 30–60%; high: 50–90%; extreme: 75–100%. To assess what proportion of total variability is due to differences between the samples analyzed, Cochran’s *Q* test was applied.

### Packages and reports

To perform diagnostic precision analyses, the following packages were used in the R statistical environment: ellipse, mada, meta, metafor, mvmeta, mvtnorm, and rmeta. A significance level of *P* < 0.05 was established, and the 95% CI were calculated. The results are reported in three decimal places. All statistical analyses and graphical representations were performed using R statistical software (version 4.1.3).

## Results

### Search results

**Figure 2:**
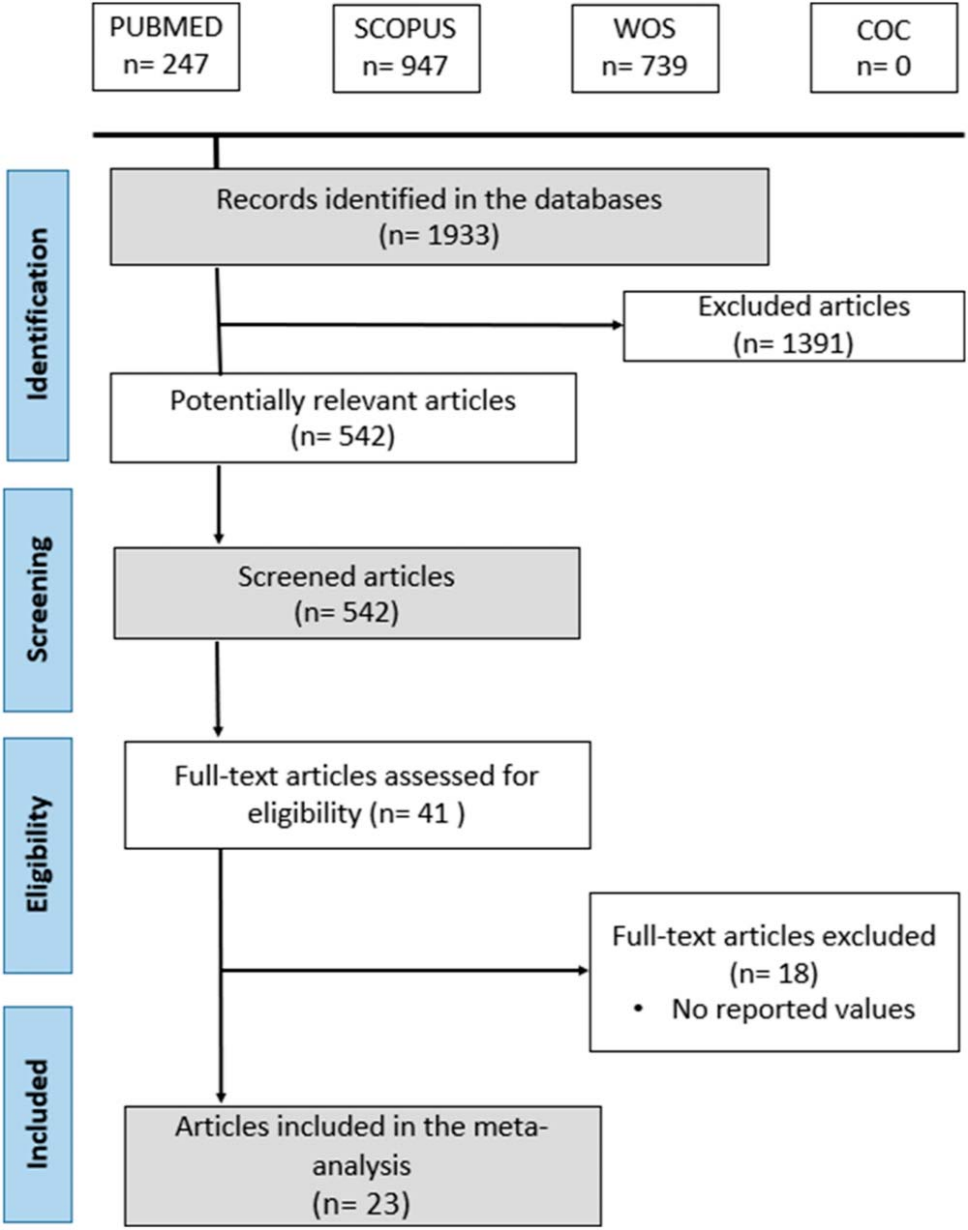
PRISMA 2020 flow diagram.

The flowchart presented in Fig. 2 follows the PRISMA 2020 guidelines and describes all included studies. The initial search across selected bibliographic databases identified 1,933 scientific articles. After excluding more than 1,300 studies, 542 articles were considered potentially relevant. Through the application of screening and eligibility criteria, the selection was further refined to 23 articles, collectively reporting 59 ML and DL models. The final sample included the model reported with the highest diagnostic performance for incorporation into the meta-analysis.

### Studies’ features

Among the selected articles, a clear trend emerges: as years progress, the number of published studies increases. This reflects the growing interest of healthcare teams in implementing these strategies to improve the diagnostic accuracy of the musculoskeletal conditions being evaluated. When grouping the articles by continent of origin, Asia emerges as the leader with the highest number of publications, reflecting significant efforts in this region. North America and Europe follow, while South America has the lowest contribution. Among the identified diagnostic methods, medical imaging was the most represented, with a total of 19 articles, focusing mainly on MRI. In addition, four new diagnostic methods were identified. Regarding musculoskeletal conditions, there was a notable interest in studying knee joint disorders, with a particular emphasis on the anterior cruciate ligament. It should be noted that, although CNN was used in a slightly higher number of articles, the distribution of the other implemented models remains relatively homogeneous. For more details, see Table 2. The following section presents the diagnostic metrics for the selected studies. See Table 3.

**Table 2:** Summary of studies on ML and DL models for ligament injury diagnosis.

Study	Reference	Country	Diagnostic method	Condition	Model	*g*	TP	FP	TN	FN
1	Astolfi *et al.* (29)	Brazil	MRI	Ankle ligament injury	Random forest	0	578	150	566	102
2	Chang *et al.* (30)	USA	MRI	ACL tear	Resnet + U-net	1	53	0	56	7
3	Cheng *et al.* (31)	China	MRI	ACL tear	SVM model 2	0	75	3	17	13
4	Fang Liu *et al.* (32)	USA	MRI	ACL tear	MRnet	1	48	2	48	2
5	Germann *et al.* (33)	Switzerland	MRI	ACL tear	DCNN	1	223	23	255	11
6	Guha Paul *et al.* (34)	Bangladesh	CT	CSL	MobileNetV2	1	199	0	200	1
7	Ito *et al.* (35)	Japan	X-ray	TPLL	YOLO v4	1	121	46	125	4
8	Jo *et al.* (36)	Korea	MRI	TPLC	InResNetV2	1	41	3	47	9
9	Kanthavel *et al.* (37)	Saudi Arabia	X-ray	Osteoarthritis	DRRL	1	93	127	372	42
10	Kim *et al.* (38)	Korea	X-ray	CPLL	ADA	0	4	1	142	28
11	Liang *et al.* (39)	China	MRI	ACL tear	ResNet	1	179	26	329	96
12	Michael R *et al.* (40)	USA	MRI	ACL tear	FS	0	49	1	150	1
13	Minamoto *et al.* (41)	Japan	MRI	ACL tear	CNN	0	46	7	43	4
14	Mouchotte *et al.* (42)	France	GNRB-MRI	ACL tear	New method	1	78	0	5	5
15	Shemesh *et al.* (43)	Israel	MRI	CPLL	CNN	0	578	102	702	14
16	Tamai *et al.* (44)	Japan	X-ray	CPLL	E-NetB2	1	219	34	209	24
17	Tedesco *et al.* (45)	Ireland	Motion sensor	ACL tear	XGB	0	2,526	1,158	1,978	562
18	Wang *et al.* (46)	China	MRI	Knee imaging	PI protocol	0	14	0	31	1
19	Whiteside *et al.* (47)	USA	Tracking data	UCL	SVM	0	78	26	77	27
20	Yingkai *et al.* (48)	China	MRI	Meniscus injury	C-PCNN	1	548	62	654	132
21	Zhang *et al.* (49)	China	MRI	ACL tear	CNN, 3D DenseNet	1	41	2	37	1
22	Zhang *et al.* (50)	China	MRI	ACL tear	CNN/MGSA	1	520	40	277	80
23	Zhu *et al.* (51)	China	Nomogram	A-spondylitis	SVM-RFE	0	77	17	91	17

**Table 3:** Performance metrics of ML and DL models for ligament injury diagnosis (29, 30, 31, 32, 33, 34, 35, 36, 37, 38, 39, 40, 41, 42, 43, 44, 45, 46, 47, 48, 49, 50, 51).

Study	Reference	SE	SP	Accuracy	F1-score
1	Astolfi *et al.* (29)	0.850	0.791	0.819	0.821
2	Chang *et al.* (30)	0.883	1.000	0.940	0.938
3	Cheng *et al.* (31)	0.852	0.850	0.852	0.904
4	Fang Liu *et al.* (32)	0.960	0.960	0.960	0.960
5	Germann *et al.* (33)	0.953	0.917	0.934	0.929
6	Guha Paul *et al.* (34)	0.995	1.000	0.998	0.997
7	Ito *et al.* (35)	0.968	0.731	0.831	0.829
8	Jo *et al.* (36)	0.820	0.940	0.880	0.872
9	Kanthavel *et al.* (37)	0.689	0.745	0.733	0.524
10	Kim *et al.* (38)	0.125	0.993	0.834	0.216
11	Liang *et al.* (39)	0.651	0.927	0.806	0.746
12	Michael *et al.* (40)	0.980	0.993	0.990	0.980
13	Minamoto *et al.* (41)	0.920	0.860	0.890	0.893
14	Mouchotte *et al.* (42)	0.940	1.000	0.943	0.969
15	Shemesh *et al.* (43)	0.976	0.873	0.917	0.909
16	Tamai *et al.* (44)	0.901	0.860	0.881	0.883
17	Tedesco *et al.* (45)	0.818	0.631	0.724	0.746
18	Wang *et al.* (46)	0.933	1.000	0.978	0.966
19	Whiteside *et al.* (47)	0.743	0.748	0.745	0.746
20	Yingkai *et al.* (48)	0.806	0.913	0.861	0.850
21	Zhang *et al.* (49)	0.976	0.949	0.963	0.965
22	Zhang *et al.* (50)	0.867	0.874	0.869	0.897
23	Zhu *et al.* (51)	0.819	0.843	0.832	0.819

### Risk of bias

**Figure 3:**
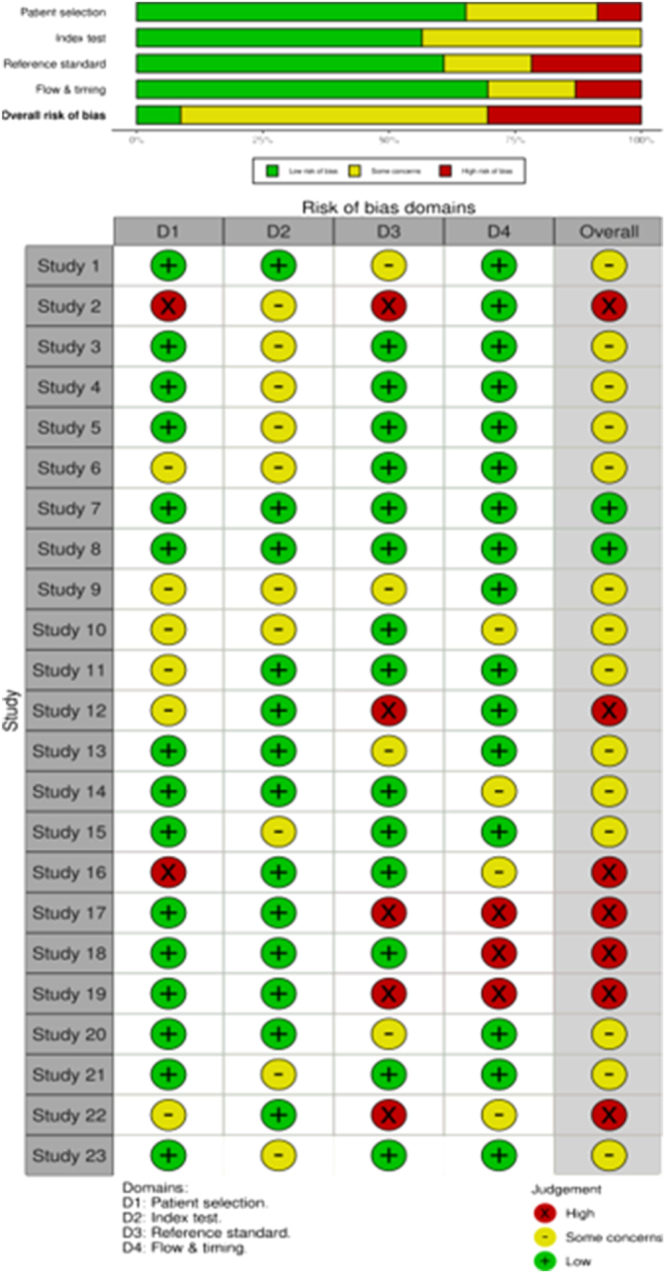
Quality assessment of included studies (29, 30, 31, 32, 33, 34, 35, 36, 37, 38, 39, 40, 41, 42, 43, 44, 45, 46, 47, 48, 49, 50, 51).

The 23 selected articles were rigorously analyzed using the QUADAS-2 tool (see Fig. 3). For further details, refer to Fig. 3. On average, seven studies exhibited a high risk of bias, with the dimension three being the most affected. In contrast, only two studies consistently demonstrated a low risk in all evaluated criteria. The remaining articles contained insufficient or unclear information, preventing a definitive assessment.

### Univariate analysis

**Figure 4:**
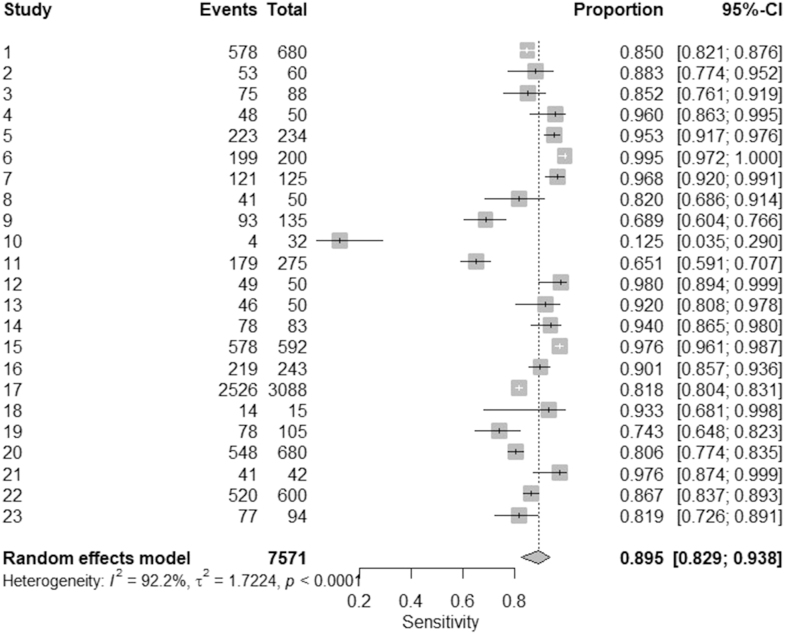
The forest plot of pooled sensitivity (29, 30, 31, 32, 33, 34, 35, 36, 37, 38, 39, 40, 41, 42, 43, 44, 45, 46, 47, 48, 49, 50, 51).

The studies were included in the meta-analysis, comprising 7,571 events. The pooled sensitivity, estimated using a random-effects model, was 0.895 with a 95% CI of 0.829–0.938, indicating a high overall diagnostic precision between studies. Sensitivity estimates in individual studies showed considerable variability, ranging from 0.125 (95% CI: 0.035–0.299) to 0.995 (95% CI: 0.972–1.000), reflecting differences in study populations, methodologies, and diagnostic criteria. Significant heterogeneity was observed between the studies (*I*^2^ = 92.2%, *τ*^2^ = 1.7224, *P* < 0.0001), suggesting that variation in sensitivity estimates is not only due to random chance but may be influenced by factors at the underlying level of the study. The high value *I*^2^ indicates substantial inconsistency, warranting further exploration through subgroup analyses or meta-regression to identify potential sources of heterogeneity. Despite this variability, the consistently high sensitivity in most studies underscores the effectiveness of the diagnostic method in diverse clinical settings. Details are shown in Fig. 4.

In the specificity analysis, it included 8,241 events. The pooled specificity, calculated using a random-effects model, was 0.926 with a 95% CI of 0.872–0.959, reflecting high general precision in correctly identifying true negatives. Specificity estimates for individual studies ranged from 0.631 (95% CI: 0.614–0.648) to 1.000 (95% CI: 0.936–1.000), indicating variability in diagnostic performance depending on the context of the study and the methodologies applied.

**Figure 5:**
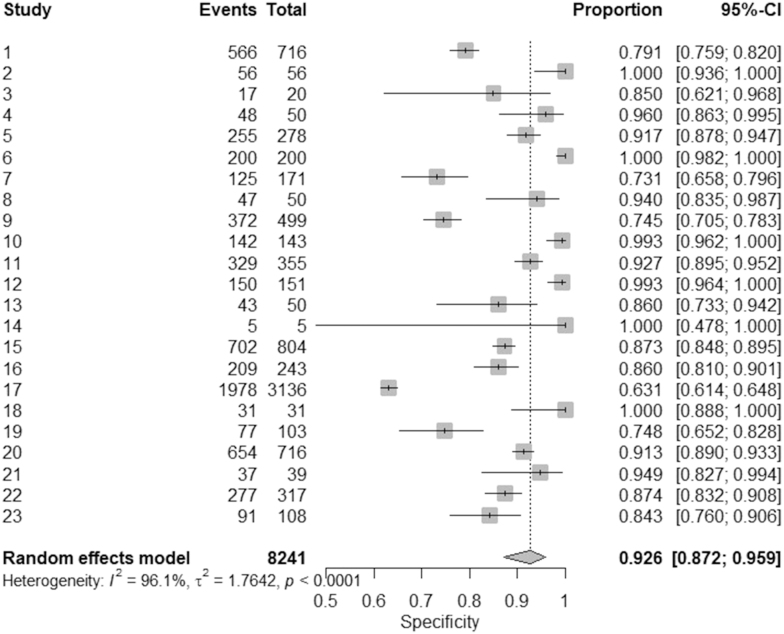
The forest plot of pooled specificity (29, 30, 31, 32, 33, 34, 35, 36, 37, 38, 39, 40, 41, 42, 43, 44, 45, 46, 47, 48, 49, 50, 51).

Substantial heterogeneity was detected between studies (*I*^2^ = 96.1%, *τ*^2^ = 1.7642, *P* < 0.0001), suggesting that differences in study design, populations, or diagnostic thresholds contributed to the observed variation. The high value *I*^2^ points to considerable inconsistency, highlighting the need for further subgroup analyses or meta-regression to identify potential sources of heterogeneity. Despite this, the consistently high specificity in most studies underscores the reliability of the diagnostic tool in diverse clinical environments. For more details, please see Fig. 5.

The analysis of the PLR yielded a mean value of 1,644.37, indicating a strong ability of the diagnostic test to confirm the presence of disease when the result is positive. The 95% CI for the PLR ranged from 73.56 to 3,215.18, reflecting considerable variability in the performance of the test between different studies or populations of patients. Although the high mean PLR suggests robust diagnostic utility, the wide CI indicates potential inconsistencies that may warrant further investigation to understand the underlying causes of this variation.

The NLR analysis showed a mean value of 0.179, indicating a high ability of the diagnostic test to rule out disease when the result is negative. The 95% CI for the NLR ranged from 0.095 to 0.263, suggesting relatively consistent diagnostic performance between studies. The low mean NLR reflects the test’s effectiveness in minimizing false negatives, supporting its reliability as a diagnostic tool in various clinical contexts.

**Figure 6:**
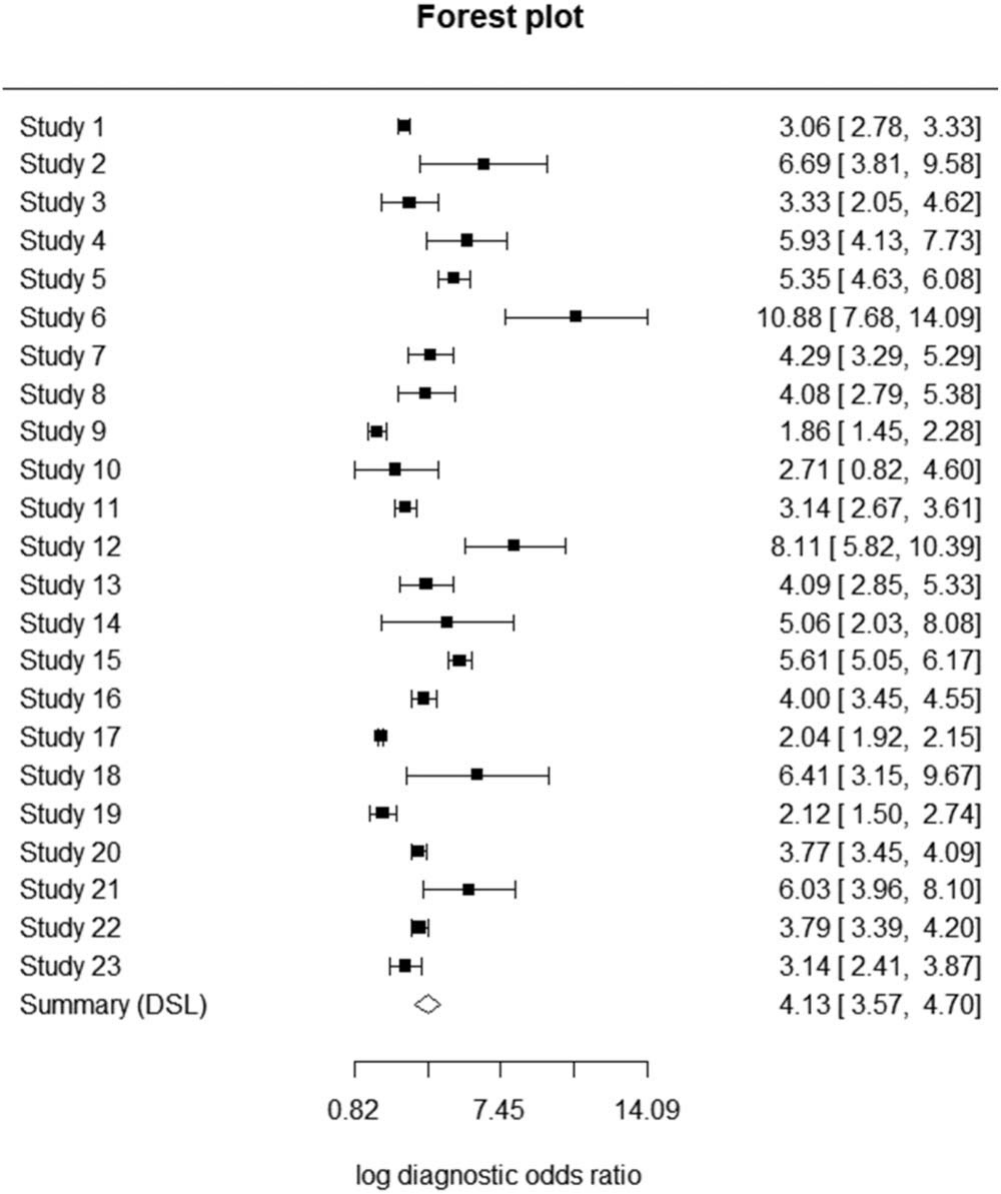
Forest plot of the log DORs (29, 30, 31, 32, 33, 34, 35, 36, 37, 38, 39, 40, 41, 42, 43, 44, 45, 46, 47, 48, 49, 50, 51).

The DOR, evaluated in 23 studies, yielded a pooled log DOR of 4.13 with a 95% CI of 3.57–4.70 (see Fig. 6). This result indicates a strong discriminatory power of the diagnostic test in differentiating between diseased and non-diseased individuals. Individual log DOR values ranged from 1.86 (95% CI: 1.45–2.28) to 10.88 (95% CI: 7.68–14.09), demonstrating variability in diagnostic performance between studies. Consistent positive log DOR values across studies suggest reliable diagnostic efficacy, although the observed range indicates that certain study-specific factors, such as population characteristics or methodological differences, may influence diagnostic precision.

### Bivariate analysis

**Figure 7:**
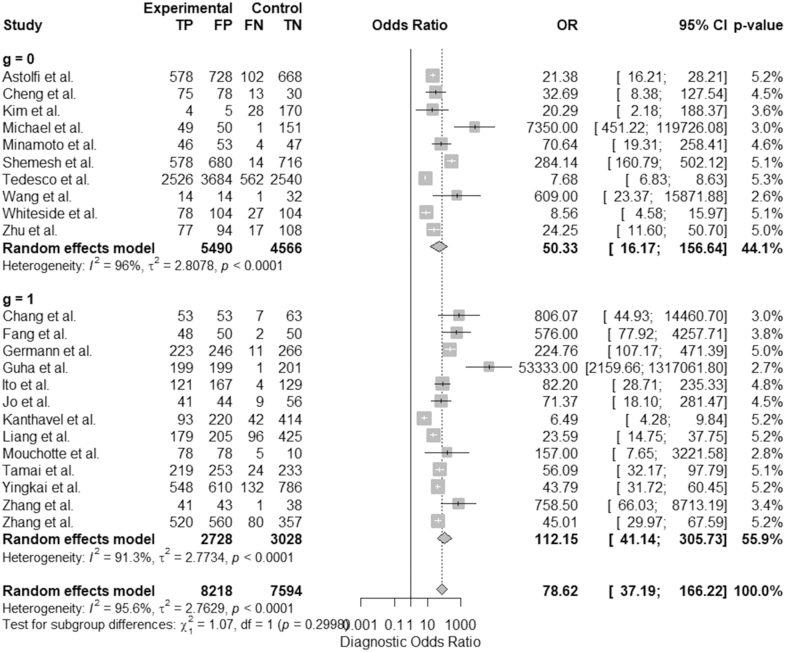
Forest plot of the odds ratios (29, 30, 31, 32, 33, 34, 35, 36, 37, 38, 39, 40, 41, 42, 43, 44, 45, 46, 47, 48, 49, 50, 51).

When analyzing subgroups based on the variable *g*, the group *g* = 0 exhibited an odds ratio (OR) of 50.33 (95% CI: 16.17–156.64) with *τ*^2^ = 2.8078 and *I*^2^ = 96%, indicating significant heterogeneity within this group. In contrast, the group *g* = 1 showed a higher OR of 112.15 (95% CI: 41.14–305.73), with *τ*^2^ = 2.7734 and *I*^2^ = 91.3%. Despite these differences, the test for subgroup differences yielded a *χ*^2^ value of 1.07 with 1 degree of freedom (*P* = 0.2998), suggesting that there is insufficient evidence to assert significant differences in odds ratios between the two subgroups. The bivariate diagnostic random-effects meta-analysis (see Fig. 7), using the REML estimation method, showed significant fixed-effects coefficients. The intercept for logarithmic transformation sensitivity (tsens) was 2.050 (SE = 0.273, *z* = 7.498, *P* < 0.0001), while the intercept for logarithmic transformation false positive rate (tfpr) was −2.164 (SE = 0.223, *z* = −9.698, *P* < 0.0001). The estimated pooled sensitivity was 0.886 (95% CI: 0.820–0.930), and the false positive rate was 0.103 (95% CI: 0.069–0.151).

**Figure 8:**
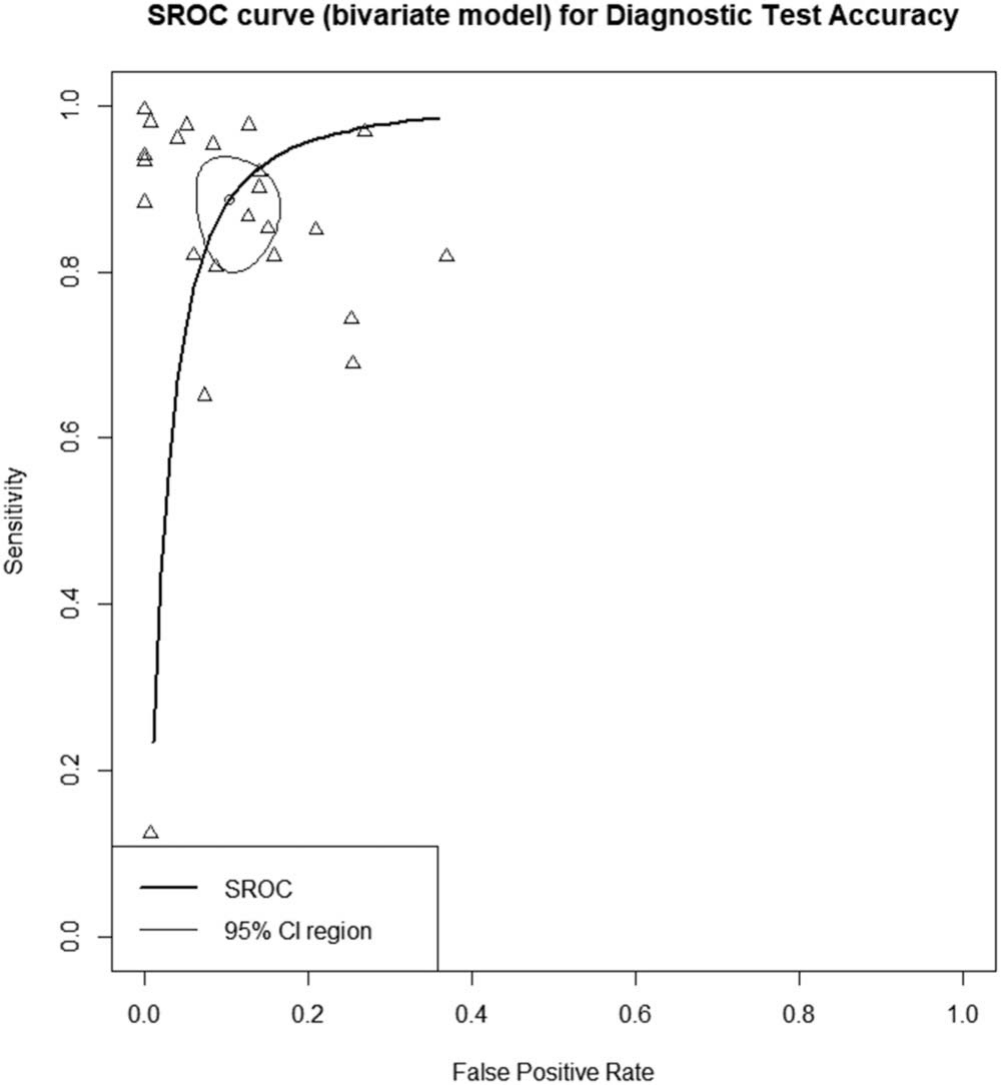
SROC curve (bivariate model) for diagnostic test accuracy.

The variance components revealed standard deviations between studies of 1.230 for the sensitivity and 0.926 for the false positive rate, with a weak negative correlation of −0.140 between them. The logarithmic likelihood of the model was 47.220, with an AIC of −84.440 and a BIC of −75.296. The area under the curve (AUC) was 0.948, indicating excellent diagnostic performance, while the partial AUC, restricted to observed false positive rates and normalized, was 0.884. For more details, please see Fig. 8.

The heterogeneity estimates varied depending on the method used. The Zhou and Dendukuri approach estimated *I*^2^ at 24.8%, suggesting low heterogeneity (53). However, the Holling sample size-adjusted approach reported heterogeneity ranging from 68% to 95.7%, while the sample size-adjusted approach showed considerably lower heterogeneity, between 2.5 and 5.2% (54). These results highlight the strong diagnostic accuracy of the model while also indicating some variability between studies, influenced by methodological approaches and sample size adjustments.

## Discussion

This study aimed to evaluate the diagnostic performance of various models of ML and DL to recognize ligament injuries in different medical imaging modalities. The findings support the clinical utility of the evaluated diagnostic tool, highlighting its high precision in identifying both positive and negative cases. Despite the variability observed among the studies, the consistency of its high sensitivity and specificity in different clinical settings reinforces its validity and applicability. However, the significant heterogeneity detected suggests that factors such as differences in study populations, methodological variations, and diagnostic thresholds may have influenced the results. In this context, further subgroup analyses, and meta-regressions are recommended to investigate potential sources of variability. Our findings support the application and usefulness of the ML and DL models in complex clinical settings.

The increasing interest of the clinical and scientific community in integrating AI-based tools into healthcare is evident and continues to grow. Recently, a consortium of 117 professionals from 50 countries reaffirmed that trusting AI in healthcare should follow 30 recommended best practices that cover its entire life cycle (55). In our study, this trend is reflected in the wide range of research utilizing AI to diagnose ligament injuries, highlighting the growing interest among specialists and healthcare teams in adopting these tools for the treatment of musculoskeletal pathology (26, 27). To our knowledge, this is the first study to simultaneously analyze such a diverse set of ML and DL models to detect these conditions while also identifying key challenges that must be addressed.

From a technical perspective, researchers are strongly encouraged to use multiple ML or DL algorithms to enhance the robustness and generalizability of their findings (56). This is achieved by simultaneously exploring and comparing different architectures, allowing their performance to be evaluated for the specific problem at hand. It is essential to recognize that no single model is universally superior; instead, the optimal choice depends on how well a model adapts to the data set and the specific requirements of the study (57). In this study, multiple articles reported the use of several models to identify ligament injuries. Although some studies used a single model (e.g. (35, 41, 43)), others tested up to five models simultaneously (36) or even six (30, 34, 45). One study (48) reported the use of nine models. This trend highlights not only the availability of various architectures for addressing the same problem but also the increasing integration of different ML and DL approaches to optimize diagnostic accuracy in diverse clinical settings. In 2021, a comprehensive guide and checklist for clinical research using AI was introduced, providing detailed recommendations on AI model design and training parameters (58). However, a key challenge remains: establishing a standardized minimum number of models required for diagnostic applications. This would help determine whether a proposed approach truly represents the best available option.

This also requires considering the computational resources necessary for data processing. More complex models demand greater computing power, often relying on graphics processing units or tensor processing units to efficiently handle large datasets and perform advanced calculations (52). In addition, these models typically require long training times, substantial storage, and significant energy consumption. Therefore, careful planning is essential to account for these factors and ensure efficient execution of the project (59). In addition, healthcare professionals must receive training not only in understanding these technical requirements but also in correctly interpreting the models used. This ensures that the results can be applied safely and effectively in clinical practice (60).

One of the key challenges in using diagnostic support methods such as ML and DL is training, validation, and testing of models (61). During training, the model learns to classify or make predictions by progressively refining its calculations. This is achieved by repeatedly analyzing a dataset with known (labeled) responses and adjusting its parameters in each iteration. The training algorithm optimizes the predicted values relative to the actual values, and by cycling the dataset multiple times, an epoch is completed, improving accuracy with each pass (62). A critical challenge in this stage is the need for researchers to report the number of epochs used. This helps determine whether a model is undertrained (underfitting) or overfitted (overfitting). Striking the right balance in the number of epochs is essential to ensure optimal predictive performance and generalizability of the results.

The validation process presents a key challenge in the implementation of the model, as it assesses performance during training without modifying the model parameters. This is done using a dataset independent of the one used for training, which is why datasets are typically divided into unequal partitions for these processes. The primary goal of validation is to evaluate the ability of the model to generalize to new data, preventing overfitting (63). A crucial aspect of this stage is the correct segmentation and reporting of the data used for validation. Ensuring a representative sample is essential to accurately capture the variability of the study problem and provide reliable performance assessments.

Finally, the testing process represents the final phase of model evaluation and serves as an external validation of the results. The main challenge at this stage is to determine whether the previously obtained results can be applied to a completely new dataset. A common issue in this phase is selecting an appropriate test set that is sufficiently diverse and representative to prevent evaluation biases (64). Ensuring a well-chosen test set is crucial for accurately interpreting diagnostic metrics and confirming that the models can be effectively applied in real clinical settings.

These three phases highlight key considerations that must be addressed when reporting the results and performance of ML and DL models. It is recommended that the authors clearly state the sample size used in each stage (65). If the sample is too small, the model may struggle to capture meaningful relationships, potentially reducing its reliability. Therefore, we suggest reporting the total number of samples and, if possible, the class distribution to ensure transparency.

In addition, it is essential to report the number of data partitions and how they are allocated. For example, specifying the percentage of data assigned to training, validation, and testing (e.g. 70% training, 15% validation, 15% testing) improves reproducibility and clarity. Ensuring that the datasets are sufficiently large and representative is crucial to achieving robust and reliable model performance.

One of the main limitations of this study is that most of the articles analyzed focused on anterior cruciate ligament injuries in the knee, likely due to its high prevalence in the population. However, this emphasis on a single structure may reduce the generalizability of the findings. Future research should consider evaluating a broader range of ligament injuries to ensure the applicability of these models in different clinical scenarios (66). Another potential limitation is the availability of sufficient studies to rigorously assess the diagnostic performance of these models. In previous research, we have emphasized the importance of having a sufficient number of articles for a comprehensive evaluation. However, since the adoption of these technologies in clinical practice has been gradual, relevant data remain limited in some cases. To address this, we have recommended the use of models as units of analysis (26, 27). In this study, we identified a large number of articles and a diverse range of reported models, which allowed us to select only those with the best performance. This selection underscores the need to standardize the evaluation criteria. Specifically, it remains unclear whether researchers should report only the best-performing model, the average of all metrics, or include both the highest and lowest-performing models to assess variability. Standardizing these practices would improve comparability and reliability in future studies.

In addition to standardization, a critical step toward real-world implementation is multicenter external validation. Most of the studies included in this meta-analysis relied on single-center data, often with homogeneous imaging protocols, which may inflate diagnostic performance. Upcoming studies must incorporate external validation across diverse institutions, imaging devices, and patient populations. This approach not only reduces the risk of overfitting but also ensures that models are generalizable and reliable in heterogeneous clinical environments. Recent methodological guidance strongly emphasizes this point, underscoring that multicenter external validation is essential for safe translation of AI into musculoskeletal practice (63, 66, 67, 68). Addressing this gap will be fundamental to moving from proof-of-concept studies to clinically deployable diagnostic tools.

The growing interest among musculoskeletal clinicians in integrating these technologies into clinical practice is evident, and their use will undoubtedly continue to expand. However, for these models to be more clinically valuable, they must be able to identify multiple structures or types of injuries simultaneously. In other words, they should support multi-label classification to detect multiple findings within the same assessment (69). A fundamental challenge in this field is not only achieving high diagnostic accuracy but also ensuring comprehensive monitoring of the structures surrounding the injury site. Enhancing these capabilities will be crucial to improving patient management and optimizing clinical decision making.

Soon, countries must establish a robust national policy on AI (70), providing clear guidelines for a regulatory and legislative framework that governs the use of patient data and information. This policy should go beyond technical and security aspects to safeguard patient rights, ensuring compliance with ethical and legal principles in the application of AI-driven technologies in healthcare. In addition, the role of scientific ethics committees must be strengthened, as they serve as key bodies for health research projects. These committees should incorporate updated guidelines to assess the implications of AI in clinical settings, particularly with regard to algorithm transparency, fairness in outcomes, and potential biases in medical decision making. Ensuring ethical AI implementation will be critical to maintaining trust and equity in healthcare innovation.

## Conclusion

This meta-analysis clearly demonstrates that the ML and DL models evaluated improve diagnostic accuracy in clinical settings, particularly when supported by imaging resources or novel diagnostic approaches for ligament injuries. Furthermore, this study highlights the growing diversity of AI-based models, reinforcing the expectation that their application in musculoskeletal pathology will continue to expand. However, the widespread adoption of these technologies will not depend solely on the interest of healthcare professionals in improving clinical performance. It will also require the participation of health system decision makers and policy makers, who must establish robust public policies to facilitate the responsible and effective integration of AI into clinical practice.

Several challenges remain to be addressed. For example, the diversity of available models requires a thorough understanding by healthcare professionals to determine which model best fits the specific clinical problem. In addition, these models must be capable of accurately distinguishing ligament injuries from other musculoskeletal pathologies, minimizing false positives and false negatives, particularly in cases with subtle findings or suboptimal image quality. To mitigate evaluation biases, it is essential to train models in diverse clinical settings to prevent both overestimation and underestimation of diagnostic performance. This requires adjusting for variability in environmental conditions, injury characteristics, anatomical differences, and other relevant factors. A key step toward improving AI-driven diagnostic strategies would be the establishment of open access data repositories by scientific societies. These repositories could facilitate the development of more robust and generalizable models, ultimately enhancing their reliability and effectiveness in clinical practice.

The findings of this study underscore the need to explore additional diagnostic modalities to identify ligament injuries. The results suggest that ML and DL methods hold promise as diagnostic support tools, particularly when integrated with conventional techniques. Their implementation has the potential to improve diagnostic accuracy while offering more cost-effective strategies in clinical practice.

Despite these advancements, several limitations were identified. First, the ability of AI models to simultaneously detect concomitant injuries within the analyzed structures remains limited, as does their capacity for multi-label classification. In addition, most of the reviewed studies have focused on detecting ligament injuries in the lower extremity, particularly ACL injuries. This narrow scope restricts the generalizability of the results and impacts the external validity of the models. Finally, the lack of standardization in reporting training, validation, and test sets remains a challenge, hindering the ability to obtain precise performance metrics. The lack of detailed sample size information for each development phase further complicates the comparative assessment of the diagnostic accuracy of the model. Addressing these limitations is crucial for advancing the reliability and applicability of AI-driven diagnostic tools in musculoskeletal medicine.

## ICMJE Statement of Interest

The authors affirm that there is no conflict of interest that could compromise the objectivity or impartiality of the reported study.

## Funding Statement

This study was not supported by any specific grant from public, commercial, or nonprofit funding agencies.

## Author contribution statement

GD was responsible for conceptualization, software, statistical analysis, and writing the original draft. GD, EV, and JV were responsible for data curation. CJ and FF were responsible for writing review. FF was responsible for supervision and editing. All authors have read and agreed to the published version of the manuscript.
